# Peripheral *N*-methyl-D-aspartate receptor localization and role in gastric acid secretion regulation: immunofluorescence and pharmacological studies

**DOI:** 10.1038/s41598-018-25753-6

**Published:** 2018-05-10

**Authors:** Iuliia Golovynska, Tatiana V. Beregova, Tatiana M. Falalyeyeva, Ludmila I. Stepanova, Sergii Golovynskyi, Junle Qu, Tymish Y. Ohulchanskyy

**Affiliations:** 1 Key Laboratory of Optoelectronic Devices and Systems of Ministry of Education and Guangdong Province, College of Optoelectronic Engineering, Shenzhen University, 518060 Shenzhen, P.R. China; 2 Institute of Biology and Medicine, Taras Shevchenko National University of Kyiv, 01601 Kyiv, Ukraine

## Abstract

The enteric nervous system (ENS) and a glutamate receptor (GluR), *N*-methyl-D-aspartate receptor (NMDAR), participate in gastric acid secretion (GAS) regulation. NMDARs are localized in different stomach cells; however, knowledge of NMDAR expression and function in the ENS is limited. In the present study, we clarified the types of stomach cells that express the NMDARs that are involved in GAS regulation. The pharmacological method of isolated stomach perfusion by Ghosh and Shild combined with direct mapping of NMDARs by fluorescence microscopy in the rat stomach was employed. By immunofluorescence labeling with an anti-NMDA-NR1 antibody, NMDARs were found to be highly expressed in nerve cells of the submucosal and myenteric plexuses in the stomach. The exact localization of the NMDARs relevant to GAS and its mechanism of action were determined by stimulating different receptors of neuronal and stomach cells using specific secretagogues for NMDA and by selectively blocking those receptors. NMDARs relevant to GAS stimulation are mainly localized in cholinergic interneurons; however, all of the nerve cells of the submucosal ganglia are involved in the stimulating process. In addition, the NMDARs in parietal cells are involved in gastric acid inhibition via influencing H_2_-histamine receptors.

## Introduction

Gastrointestinal (GI) diseases are the leading cause of morbidity and have a great impact on life^1–3^. Stomach disorders of the acid-base balance and various pathogens^4,5^ can cause many lesions, increasing the probability of triggering cancers^6–8^. Hypoacidity of the gastric juice is a dangerous state, which leads to dysbiosis, putrefactive processes, suppression of proteolytic enzyme activation and stomach cancer^9^. On the other hand, hyperacidity of gastric juice between meals is one cause of ulcers^10^. However, in addition to diseases, the physiology and biochemistry of GI tract organs are intensively investigated and are key to the development of effective therapeutics and drugs^11^.

Gastric acid secretion (GAS) is regulated by neuronal, paracrine and endocrine mechanisms. At present, the influence of the central nervous system (CNS) on GI function is under intense studied. Glutamate (Glu) receptors (GluRs) are localized in the nucleus tractus solitarii and play an important role in neurotransmission^12^. Previous research on the role of the CNS GluRs in GAS regulation has concluded that ionotropic GluRs (iGluRs) are involved in the regulation of secretion. However, their activation affects both GAS stimulation^13^ and inhibition^14^. Different types of GluRs act in opposite ways on GAS. Namely, Glu binds to iGluRs and metabotropic receptors (mGluRs)^15^. iGluRs, such as *N*-methyl-D-aspartate (NMDA), α-amino-3-hydroxy-5-methyl-4-isoxazole-propionate (AMPA) and kainite receptors, are ligand-gated calcium and potassium ion channels^16^.

The enteric nervous system (ENS) can regulate GI motility and secretion regardless of the extrinsic sympathetic and parasympathetic input. An abundant expression of GluRs in the peripheral tissues^17,18^ indicates a more extensive role for Glu as a neurotransmitter. In regard to the ENS, both AMPA and NMDA receptor (NMDAR) subunits have been detected on neurons in both the submucosal and myenteric plexuses by means of fluorescent immunohistochemistry^12,18^. In fact, NMDARs have been found in most cells of the ENS^12,18–20^. In the stomach, they are expressed in parietal and surface cells and moderately expressed in chief cells^19^.

However, the role of NMDARs in GAS regulation has been insufficiently investigated, and the existing data are contradictory^13,14^. Furthermore, it is unknown in which type of stomach cells the NMDARs relevant for GAS regulation are expressed. New insights are needed for understanding the GAS regulating mechanisms and role of NMDARs in the GI tract overall that might allow for targeted selective treatment of stomach diseases^2,4,5^ and oncology^6–8^. The significance of GluR research has increased since the discovery that GluRs and, especially, NMDARs are involved in the tumor suppression mechanism^21,22^ and may be affected by light or so-called low-level laser therapy^23^.

In the present study, we clarified the types of stomach cells that express the NMDARs that are involved in GAS regulation. The pharmacological method of isolated stomach perfusion by Ghosh and Shild, combined with direct mapping of NMDARs in rat stomach sections by optical microscopy (immunohistochemistry and fluorescence microscopy using confocal laser scanning) were employed. First, we mapped the NMDARs across the entire width of the gastric wall from the muscular layer to the end of the stomach villus. Then, using pharmacological methods, the NMDAR regulation of GAS was investigated.

## Results

### NMDAR mapping with immunohistochemistry and fluorescence microscopy

**Figure 1:**
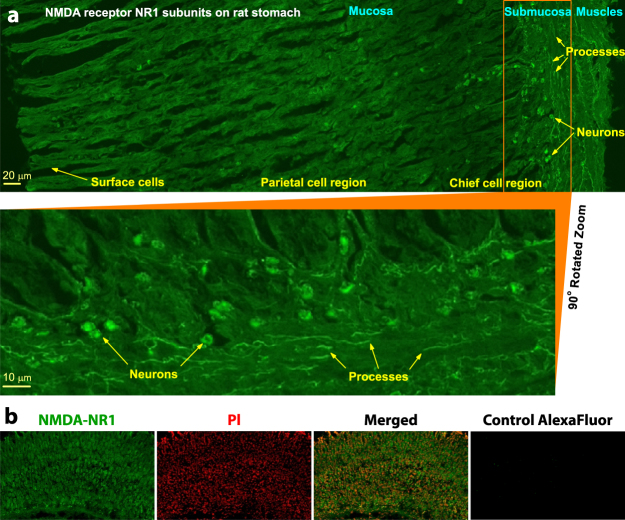
(**a**) NMDAR NR1 subunit expression in rat stomach whole wall cross sections mapped by means of immunofluorescence labeling with anti-NMDA-NR1 antibody together with Alexa Fluor 488 secondary antibody and confocal laser scanning fluorescence microscopy. (**b**) Epifluorescence microscopy of the stomach slice double-stained with anti-NMDA-NR1 antibody with Alexa Fluor 488 secondary antibody and propidium iodide (PI) along with the negative control slices prepared by staining with Alexa Fluor 488 secondary antibody only.

To directly visualize NMDARs, the primary NMDA-NR1 subunit expression in rat stomach whole wall cross sections was assessed by means of confocal laser scanning fluorescence and epifluorescence microscopy (Fig. 1a,b). For epifluorescence analysis, the cell nuclei were also stained by propidium iodide (PI) to assess the arrangement of NR1-positive-labeled cells in respect to all cells.

Certain cells and tissues may have inherent biological properties resulting in background or non-specific staining that could lead to a misinterpretation of the results. We eliminated non-specific binding and background staining and prepared a negative control (Fig. 1b) by staining the slices with only the Alexa Fluor 488 secondary antibody which exhibits minimal cross-reaction with sample proteins. The negative control, which shows the absence of fluorescence at the same imaging parameters, proves that the immunofluorescence staining by the NMDA-NR1 antibody was specific.

Most of the representative confocal fluorescence images consisted primarily of the mucosa (Fig. 1a). This stomach wall layer comprises various secretory cells such as the surface, parietal, and chief cells that are involved in the GAS mechanism^18–20^. All of the mucosa cells were labeled by the NMDA-NR1 antibody. The epifluorescence images confirm this, as all of the cells were double-labeled by the NMDA-NR1 antibody in the membrane and by PI in the cell nucleus (Fig. 1b). Therefore, all secretory cells of the stomach contain NMDARs. Such an expression of NMDARs coincides with other immunohistochemistry studies of the stomach mucosa^19,24^, in which NMDARs were shown to be expressed in the surface, parietal and chief cells.

Beneath the basal membrane, the submucosa layer comprises neurons that regulate the process of gastric juice excretion by secretory cells^18^, and it is known that GluRs^12,18,25^ and, in particular, the NMDA-NR1 subunit^20,24,26^ is highly expressed in the ENS nerve cells of the myenteric and submucosal plexuses. In Fig. 1a, neurons of the submucosa that are most intensely stained for NMDA-NR1 lie between other stomach cells. The muscular layer also contains a variety of nerve cells with long processes permeating each muscle fiber. The confocal fluorescence image clearly visualizes the fine structure of the ENS in the stomach section (see the enlarged image of the submucosa region in Fig. 1a).

Thus, NMDARs were found to be expressed in secretory cells of the stomach mucosa and were abundantly expressed in nerve cell bodies and their processes in the submucosal and myenteric plexuses. However, knowledge of NMDAR expression and function in the ENS is limited, and it is not known in which types of stomach cells the NMDARs relevant for GAS regulation are localized. Therefore, we carried out pharmacological and physiological experiments to determine the functions and distinct physiological localization of NMDARs in the stomach.

### NMDA and carbachol influences on basal GAS

**Figure 2:**
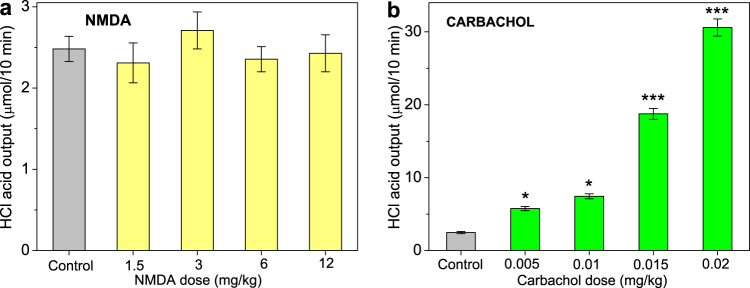
The effect of (**a**) NMDA and (**b**) carbachol at various doses on GAS compared to control basal GAS in rats following isolated stomach perfusion as described by Ghosh and Shild. The data are presented as the mean ± SD (*N* = 11). *P < 0.05, ***P < 0.001 compared with controls indicate a statistically significant difference (Student’s t-test). The effects of the drugs are evaluated by a statistically significant difference in relation to the basal acid secretion output.

Figure 2a displays the gastric acid output with NMDA treatment compared to the control basal acid output (mean ± SD) in each series of eleven independent experiments (*N* = 11). To determine the effect of NMDA on basal GAS, NMDA was injected at various doses. Tsai *et al*.^27^ reported that NMDA at doses up to 5 mg/kg did not affect basal GAS in rats. Taking this into account, for the first experiments, we tested a dose of 3 mg/kg. At this dose, NMDA did not statistically influence the acid output. NMDA at doses of 1.5, 6 and 12 mg/kg also did not demonstrate any effect. So, NMDA at all of the doses studied did not result in spontaneous GAS.

We conducted a dose-response study to determine the optimal carbachol dose that can stimulate GAS in rats and cause an effect that is equal to half of the maximum possible effect (Fig. 2b). We found that carbachol stimulates GAS in a dose-dependent manner at doses ranging from 5 to 20 µg/kg (*P* < 0.05 and *P* < 0.001, respectively). A further increase in the dose caused animal death. The optimal carbachol dose has been determined to be 10 µg/kg.

### NMDA influence on stimulated GAS

**Figure 3:**
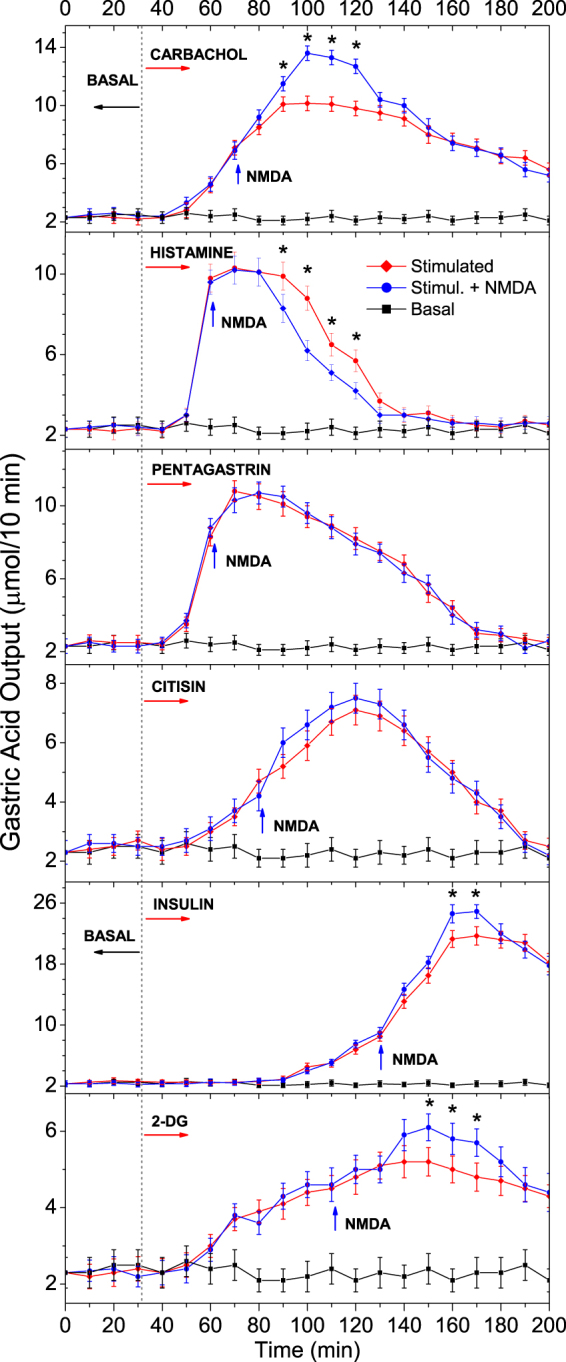
NMDA effect on GAS induced by the secretagogues carbachol, histamine, pentagastrin, cytisine, insulin, and 2-DG measured following isolated stomach perfusion as described by Ghosh and Shild. Basal acid output is presented for comparison. The data are presented as the mean ± SD (*N* = 11). The NMDA effect was evaluated by a statistically significant difference in relation to the secretagogue output, *P < 0.05 indicates a statistically significant difference (Student’s t-test).

The main series of experiments was devoted to the effect of NMDA on GAS stimulated by different secretagogues such as carbachol, histamine, pentagastrin, cytisine, insulin, and 2-deoxy-D-glucose (2-DG) (Fig. 3). The mechanism of action of these secretagogues on GAS has been well studied, while the co-action of NMDA together with each of those secretagogues remains undetermined. It should be mentioned that NMDARs can be activated only with membrane depolarization caused by receptor stimulation^28^. Hence, to determine the exact localization of the NMDARs relevant to GAS, different receptors of neuronal and stomach cells were consecutively stimulated by specific agonists. First, HCl release induced by stimulants was measured, then, after injecting NMDA, the acid output dynamics were again measured.

Carbachol is a synthetic analog of the neurotransmitter acetylcholine (ACh); however, carbachol can act for nearly 2 hr. Carbachol was chosen owing to the fact that ACh is one of the main mediators in GI functioning, and vagal GAS stimulation occurs via ACh action on nicotinic (nAChRs) and muscarinic ACh receptors (mAChRs). In our experiments, carbachol alone significantly increased HCl output (*P* < 0.05); then, with carbachol and NMDA co-stimulation, NMDA further increased HCl output (*P* < 0.05).

Histamine and pentagastrin (acting as gastrin) are powerful peripheral HCl secretagogues. Both of these secretagogues, acting alone, significantly stimulated HCl output (*P* < 0.05); however, NMDA strongly reduced histamine-induced GAS (*P* < 0.05), while an effect of NMDA on pentagastrin-induced secretion was not observed.

Cytisine is a partial nAChR agonist^29^. Cytisine caused an apparent activity for 2 hr (*P* < 0.05), while NMDA injected after cytisine caused no effect.

An insulin*-*stimulating mechanism in GAS is associated with a decrease in blood glucose levels (insulin is a GAS regulator through the CNS). It was found that insulin caused a strong secretory activity of the stomach (*P* < 0.05). Then, a statistically significant difference (*P* < 0.05) was detected between the NMDA-induced acid output compared to the insulin-induced output.

2-DG (2-deoxy-D-glucose) is a synthetic analog of glucose^30^. In another study, 2-DG was selected to re-examine its action on glucose-sensitive neurons. As with insulin, the effect of co-stimulation was weak. It was found that 2-DG caused a persistent GAS stimulation (*P* < 0.05) similar to insulin, but with a shorter latency period. NMDA was injected after 2-DG injection, and a statistically significant (*P* < 0.05) effect of NMDA on HCl release was detected (Fig. 3).

As NMDA has the strongest effect on carbachol-induced GAS, and as carbachol excites numerous receptors in the stimulation process, further investigations focused on carbachol-induced GAS.

### NMDA effect on GAS stimulated by carbachol under vagotomy

**Figure 4:**
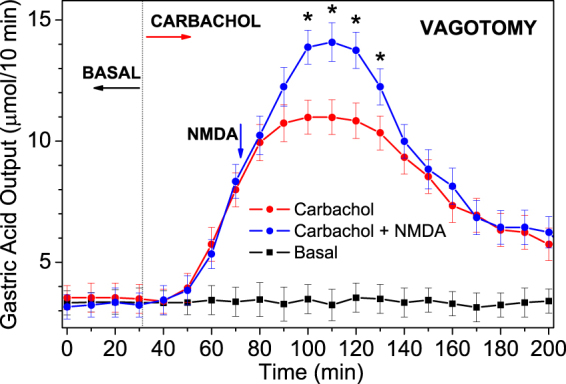
NMDA effect on carbachol-induced GAS under the conditions of bilateral cervical vagotomy measured following isolated stomach perfusion as described by Ghosh and Shild. The data are presented as the mean ± SD (*N* = 11). The effects of the antagonists and NMDA are evaluated by a statistically significant difference in relation to carbachol output, *P < 0.05 indicates a statistically significant difference (Student’s t-test).

Since D-aspartate can penetrate the blood-brain barrier^31^, we conducted a series of experiments under bilateral cervical vagotomy to terminate GAS regulation by the CNS. It was established that basal acid output 1 hr after vagotomy was 3.12 ± 0.34 µmol/10 min (Fig. 4), while basal output in intact rats was 2.28 ± 0.15 µmol/10 min (*P* < 0.05). Bilateral cervical vagotomy was shown to stimulate gastrin secretion and gastrin-induced GAS, owing to the termination of gastrin release blockade by the CNS^24^. In the next experiments under vagotomy, carbachol and NMDA caused strong secretory actions on GAS (Fig. 4); however, the results coincided with the results under the conditions of an intact nervous system, with a statistically significant difference (Fig. 3). Watanabe *et al*.^24^ showed an increase in the NMDAR number in the whole stomach using immunohistochemical and biochemical approaches 2 days after the vagotomy operation. This could potentially enhance the NMDA effect many hours after vagotomy, as the GluR synthesis is a process lasting hours, e.g., nearly 1 day in the case of mGluRs^32^. Our experiments started only 1 hr after vagotomy. This can be the most probable reason for the absence of an enhanced NMDA effect. Experiments that are performed under vagotomy clearly show that NMDA-induced acid release is solely the result of its stimulus on peripheral NMDARs.

### NMDA effect on carbachol-induced GAS under receptor blockade

**Figure 5:**
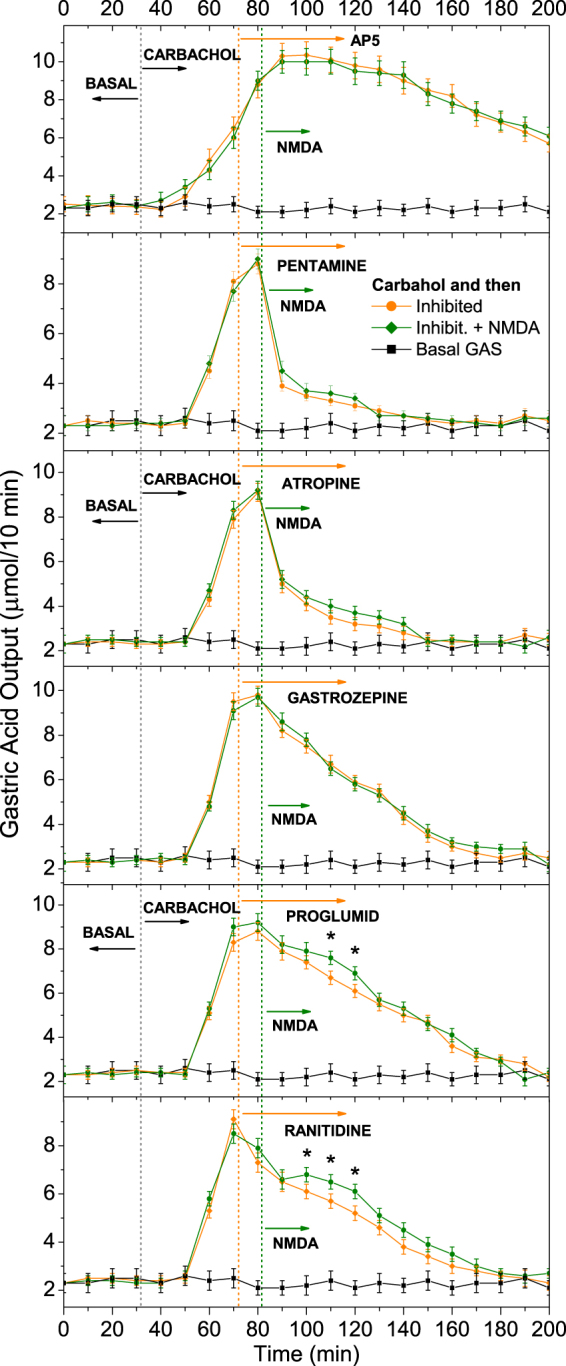
NMDA effect on carbachol-induced GAS with blockade of NMDAR by (AP5), nAChRs (pentamine), mAChRs (atropine) and m_1_AChRs (gastrozepine) along with gastrin/CCK (proglumide) and H_2_-histamine receptor (ranitidine). The basal acid output is presented for comparison. The HCl output was measured following isolated stomach perfusion as described by Ghosh and Shild. The data are presented as the mean ± SD (*N* = 11), *P < 0.05, compared with the output under the inhibitor action indicates a statistically significant difference (Student’s t-test).

In the next experiments, the depolarization of the nerve cell membrane was caused by prior carbachol stimulation. To elucidate some aspects of NMDAR functions, the study of NMDA action was conducted under the influence of selective receptor antagonists (Fig. 5). The following injection sequence was carried out: (i) carbachol after control basal GAS measurements, (ii) inhibitor 40 min later, and (iii) NMDA 10 min after the inhibitor injection.

AP5 (DL-2-amino-5-phosphonovaleric acid), a selective NMDAR antagonist that competitively inhibits the Glu binding site of NMDAR was used to demonstrate the specificity of NMDA effects. After the AP5 injection, carbachol-induced GAS was equal to that of carbachol action alone, and NMDA did not stimulate HCl release.

Pentamine, atropine and gastrozepine, which are nonselective nAChR, mAChR and m_1_AChR blockers, respectively, were selected to examine the NMDA effect on blockade by these receptors. After the inhibitor injections, carbachol-induced GAS was strongly suppressed by the antagonists, and NMDA stimulation was fully eliminated.

Proglumide and ranitidine can block gastrin/cholecystokinin (CCK) or H_2_-histamine receptors on parietal cells, respectively, resulting in GAS reduction. Both inhibitors reduced carbachol-induced GAS, while NMDA weakly increased HCl output (*P* < 0.05).

## Discussion

NMDARs were found to be highly expressed in nerve cell bodies and their processes in the submucosal and myenteric plexuses and also on surface, parietal and chief cells of the stomach mucosa. These observations coincide with the data reported elsewhere^18–20,24–26^. During the pharmacological study, we focused on the involvement of NMDA-type GluRs in GAS regulation.

The specificity of the NMDA effect, thereby the proof of the Glu physiological role, was demonstrated by blocking NMDARs with the AP5 competitive antagonist. After AP5 injection, NMDA stimulation on the background of carbachol GAS caused no additional HCl release, as the co-induced HCl output statistically duplicated the output after carbachol stimulus alone.

In the experiments investigating NMDA influence on stimulated GAS, different secretagogues, such as carbachol (acting as ACh), histamine, pentagastrin (acting as gastrin), cytisine (acting as nicotine), insulin, and 2-DG (acting as glucose) were selected. Their use in experiments allows for the analysis of secretory processes at different phases of gastric secretion due to the involvement of these substances in a long chain of chemical reactions that are responsible for GAS.

**Figure 6:**
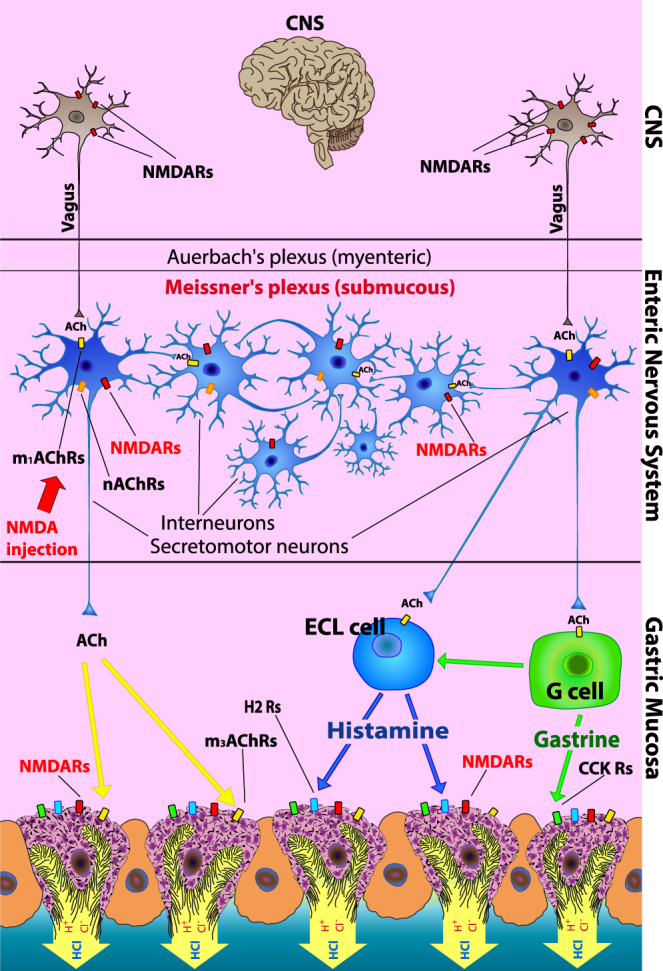
Schematics illustrating the GAS regulation mechanism and involvement of NMDARs in this process. This general scheme is based on data from published literature (see Refs. in the Discussion). Our data regarding the localization of the NMDARs responsible for GAS stimulation and inhibition (represented in Meissner’s plexuses and parietal cells, respectively) are highlighted in red.

The main nerve that regulates GAS is the vagus nerve^33^ and vagal stimulation is mediated by ACh. The neuron presynaptic membrane releases ACh, which acts on postsynaptic nAChRs and m_1_AChRs in secretomotor and sensory neurons and interneurons in the intramural ganglia of Meissner’s plexus^34^. ACh, which is released from postganglionic nerve fibers, stimulates m_3_AChRs of parietal cells, thus initiating HCl release^35^. Carbachol, which is a synthetic analogue of ACh, affects parietal cells by the same pathway, stimulating all cholinergic cells of Meissner’s plexus. ACh not only stimulates the activity of parietal and chief cells but also causes gastrin secretion by G-cells of the stomach antral region. Gastrin is the strongest stimulant of parietal cells and, to a lesser extent, chief cells, acting through CCK receptors. Gastrin release is enhanced in the presence of amino acids and dipeptides, and with moderate stretching of the stomach antrum. This causes excitation of the sensory link-neurons of the ENS peripheral reflex arc and activation of G-cells through interneurons. Additionally, ACh increases the enterochromaffin-like cell histidine decarboxylase activity, leading to a histamine concentration increase in the gastric mucosa. Histamine is a stimulant of acid release in the stomach, and it acts on the H_2_-receptors of parietal cells, being crucial for the secretory activity of these cells^36^. Histamine also stimulates the secretion of gastric acid proteinases; however, the sensitivity of zymogen cells to histamine is low due to a low density of H_2_-histamine receptors on the parietal cell membrane. Carbachol-, histamine- and pentagastrin-induced GAS is most cognate to the gastric phase^37^. All of these mechanisms are illustrated in Fig. 6.

It has been shown that NMDA increases carbachol-induced HCl output by 44 ± 9% (*P* < 0.05), which is similar to the carbachol-induced secretion. GAS stimulation by other drugs increased weakly or not at all after co-stimulus by NMDA. In view of the mechanisms of ACh action, we can consider the mechanisms by which NMDA acts on a carbachol background to stimulate HCl secretion. Carbachol activates m_1_AChRs and nAChRs localized in the intramural ganglia of Meissner’s plexus^34^ and causes membrane depolarization. NMDARs are also located in Meissner’s plexus^12,18^, and the depolarization together with the NMDA effect causes NMDAR opening^28^. This results in the additional stimulation of neurons and additional ACh release from the presynaptic membrane. In turn, ACh-activated m_3_AChRs stimulate parietal cells providing additional HCl release^35^. Furthermore, in our experiment with vagus nerve transection, the co-action of NMDA and carbachol-induced GAS has shown an increase in output that is comparable to that of intact rats, thus identifying the ENS localization of the NMDARs responsible for GAS.

The peripheral secretagogues histamine and pentagastrin sufficiently stimulated HCl output on their own; however, strong disparities in NMDA action were observed. In the case of peripheral injections, NMDA reduced histamine-induced GAS, while an effect of NMDA on pentagastrin-induced secretion was not observed. These results correlate with other studies. In particular, Tsai *et al*. have shown that, in the case of peripheral injections, L-Glu and NMDA reduced GAS stimulated by histamine^25^, whereas L-Glu had no effect on acid secretion induced by pentagastrin^38^. However, the release of endogenous Glu in the brainstem leads to an inhibition of the secretion induced by peripheral secretagogues such as pentagastrin^14^. Other researchers have shown that Glu affected basal GAS by injecting Glu directly into the CNS^39^. These disparities in the peripheral secretagogue actions can be explained by the fact that Glu and NMDA can interact with different structures in the nervous system. Taking into account the mechanisms of histamine action, it can be concluded that the NMDARs of parietal cells are involved in gastric acid inhibition through the action of H_2_-histamine receptors. The existence of NMDARs in parietal cells has been determined by means of fluorescence immunohistochemistry^19^; however, the role of these receptors has not been fully clarified. In the case of pentagastrin action on the CCK receptor of parietal cells, NMDARs are not activated for some reason. One of the possible mechanisms may be an insufficient membrane depolarization for NMDAR activation. We can thereby assume that NMDARs are selective parietal cell protectors for the histamine secretory pathway at the least, and, clearly, NMDARs in parietal cells are not involved in GAS stimulation. This coincides with the data of our studies devoted to the NMDA influence on carbachol-induced GAS under conditions of gastrin/CCK and H_2_-histamine receptor selective blockade. Proglumide or ranitidine reduced carbachol-induced GAS, while NMDA weakly increased acid output in both cases. So, the NMDA effect on carbachol-induced GAS highly depends on the functional state of parietal cells, and, when one of the triggered mechanisms of acid secretion as gastrin or histamine is suppressed, the other is active.

To investigate the role of nerve cells belonging to Meissner’s plexus in the process of NMDA-induced GAS, secretomotor neurons were excited by insulin or 2-DG and further injection of NMDA. GAS stimulation by insulin and 2-DG is similar to the GAS cephalic phase^37,40^. Insulin excites glucose-sensitive neurons in the lateral hypothalamus, where the neuroexcitation is transmitted from the solitary tract nucleus to that of the vagus nerve, triggering ACh neurotransmission. 2-DG, unlike insulin, does not cause lateral hypothalamus excitement and acts directly on neurons of the vagus nerves nucleus, stimulating parietal cells through secretomotor neurons^30^. The NMDA effect on insulin/2-DG-induced GAS indicates that the NMDARs in the secretomotor neuron postsynaptic membrane^41^ are involved in GAS stimulation. Likely, this participation of secretomotor neurons is minor and is mostly dedicated to the transfer pathway because, taking into account the high expression of NMDAR in secretomotor neurons^41^, the action of NMDA would be comparable or higher than that caused by carbachol. However, a relative increase in the insulin/2-DG and NMDA co-induced acid output was much lower than that of the carbachol and NMDA co-stimulation. The latter two molecules act simultaneously on nAChRs and m_1_AChRs in all nerve cells of Meissner’s plexus ganglia, highly exciting those receptors. It can thereby be concluded that most neuroexcitation under the action of NMDA occurs outside secretomotor neurons, namely, in interneurons. Subsequently, secretomotor neurons are excited by interneurons via Ach release, which then excite parietal cells. Our assumption is partially confirmed by the published evidence that all interneurons in the ENS are of a cholinergic phenotype^42^, so they can effectively participate in ACh neurotransmission that is highly enriched by NMDARs^41^.

Our new data regarding the localization of the NMDARs involved in GAS stimulation (in Meissner’s plexus) and inhibition (by parietal cells) complement previously known data about the GAS mechanism and allow us to detail this mechanism as shown in Fig. 6. New insights about GAS stimulatory mechanisms under NMDA co-action have also been obtained from other experiments. Nicotine and cytisine reveal different effects on the GI tract, being stimulants of nAChRs in cells of Meissner’s plexus^43^. When we injected NMDA after cytisine, the secretion level did not change. This result suggests an insufficient membrane depolarization for NMDAR activation when exciting only nAChRs in the ganglia. This predication was also confirmed by the experiment of mAChR blockade by atropine and m_1_AChR blockade by gastrozepine over the background of carbachol-induced acid release. In such a configuration of drug injection, only nAChRs were excited, and NMDA did not change HCl output. Moreover, it has been reported^13^ that GAS stimulated by kainate and NMDA was completely blocked by systemic atropine injection. A similar elimination of an NMDA influence was observed with the experiments of nAChR blockade by pentamine, which also point to an insufficient membrane depolarization in the case of m_1_AChR excitation in the ganglia.

Thus, for the activation of the NMDARs located in the submucosal Meissner’s plexus ganglia, the simultaneous activation of both m_1_AChRs and nAChRs is a critical necessity, which is naturally carried out by ACh released from nerve cell bodies^44^. The mechanism of this process can be assumed to be as follows. NMDAR channels are blocked by external magnesium (Mg^2+^)^45^; Ach, acting through m_1_AChRs and nAChRs, causes membrane depolarization that is sufficiently high for Mg^2+^ displacement from NMDAR channels. Conceivably, iGluRs and mGluRs are closely interrelated in the stomach^46^, as AMPARs are also abundantly expressed in secretory cells and in neurons of the ENS^18,47^. So, when Glu is present, AMPARs are activated, which also leads to membrane depolarization and NMDAR activation^46^. After stimulation through the glutamatergic pathway, NMDARs are excited by Glu contained in axons of the stomach wall. These axons originate from neuronal cell bodies localized in the myenteric and submucosal plexuses^48^. It is known that cholinergic and glutamatergic systems have a close interaction because varicose and smooth Glu-immunoreactive nerve fibers, which often contain choline acetyltransferase (ChAT) and vesicular ACh transporter, are closely adjacent to the bodies of neuron subpopulations in the myenteric and submucosal plexuses. In the stomach ENS, glutamatergic neurons express ChAT and the synapses have a Glu capture system with a high affinity^18^.

## Conclusions

Combining immunohistochemistry and isolated stomach perfusion by Ghosh and Shild, a detailed analysis of ENS localization of the NMDARs involved in GAS regulation was performed. The use of fluorescence microscopy offered a precise spatial mapping of NMDARs, which were found to be moderately expressed in secretory cells of the stomach mucosa and were also highly expressed in neurons of the submucosal and myenteric plexuses. A pharmacological method allowed us to determine that the NMDARs relevant for GAS stimulation are mainly localized in the cholinergic interneurons; however, all nerve cells of the submucosal Meissner’s plexus ganglia are involved in the stimulation process. Additionally, the NMDARs in parietal cells are involved in gastric acid inhibition via an action on H_2_-histamine receptors. We have assumed that NMDARs are a selective parietal cell protector for the histamine secretory pathway. Furthermore, it has been determined that, for the activation of NMDARs in the submucosal Meissner’s plexus ganglia, a simultaneous activation of both m_1_AChRs and nAChRs is a critical necessity.

## Methods

### Animals

The animal studies were carried out at Taras Shevchenko National University of Kyiv, Ukraine, using Wistar female rats, 4–5 months old, weighing 180–200 g, in strict accordance with the recommendations about the general ethical principles of animal experiments in the “Guide for the Care and Use of Laboratory Animals” by the National Institutes of Health and the experimental protocols approved by the Bioethics Committee of Animal Experiments in the ESC “Institute of Biology and Medicine” at Taras Shevchenko National University of Kyiv, Ukraine from 09.02.2015. The rats were maintained in collective cages under standard controlled conditions on a 12 hr light/dark cycle and fed standard rodent chow and water ad libitum.

### Immunohistochemistry and fluorescence microscopy

For fluorescence microscopy, a protocol similar to that described elsewhere^19,20^ was used. Immediately after collection, the stomach was fixed in 10% neutral buffered formalin for 48 hr. Then, dehydration was carried out, which involves immersing the specimen in increasing concentrations of alcohol to remove moisture and formalin from the tissue. An organic solvent, xylene, was used to remove the alcohol. The specimens were infiltrated with paraffin wax. Then, 20 µm thick sections of tissue were cut. Once cut, the tissue ribbons were carefully transferred to a warm water bath. The slides were labelled and then allowed to dry upright at 37 °C for several hr.

For fluorescence/confocal microscopy, the slides were incubated with anti-NMDA-NR1 polyclonal antibody (NMDAR1 PA5–34599 from ThermoFisher Scientific, China). The sections were stained with anti-NMDA-NR1 antibody according to the manufacturer’s instructions. Briefly, the tissue sections on slides were probed for 24 hr at 4 °C in a humidified chamber with rabbit polyclonal anti-NMDAR1 at an antibody concentration of 1–2 µg/ml diluted in 50 mM Tris-HCl, pH 7.4, containing 1.5% NaCl, 0.3% Triton X-100 (TBST) and 4% normal goat serum. Then, the Goat anti-Rabbit IgG (H + L) Highly Cross-Adsorbed Secondary Antibody, Alexa Fluor Plus 488 (ThermoFisher Scientific, China) was applied at a dilution of 1:1000 for 1 hr at room temperature.

To prevent a non-specific binding and background staining, before applying primary NMDA-NR1 antibody, the slices were incubated with 0.3% Triton X-100 and 4% normal goat serum for 1 hr following a standard method^49^. One of these reagents was also contained in the diluent of primary and secondary antibodies. The addition of non-ionic detergents including 0.3% Triton X-100 can also reduce non-specific interactions.

Following staining, a cover slip was mounted over the tissue specimen on the slide using optical grade glue to help protect the specimen. The labeled slices were imaged with a Leica TCS SP2 (Leica Microsystems, Germany) confocal laser scanning microscope (using excitation with 488 nm laser line and fluorescence detection in 500–560 nm range).

For the preparation of the negative control, the slices were treated with 0.3% Triton X-100 and 4% normal goat serum and Alexa Fluor 488 secondary antibody at a dilution of 1:1000 for 1 hr at room temperature. For the comparison of the NR1-stained slices with the negative control and also for the visualization of cell nuclei, additional slices were double-stained with NR1 antibody, following the method described above, and then with PI from ThermoFisher Scientific. Since the samples were fixed using 70% ethanol, the membrane has become permeable for PI, allowing nuclear staining. PI was used at a concentration of 2 µg/ml for 5 min at room temperature.

Finally, the double-stained and negative control stomach sections were inspected at the same parameters of fluorescent imaging, employing a Nikon Eclipse Ti-U inverted microscope equipped with a Nikon Digital Sight DS-Fi2 camera (Nikon, Japan) at 10x magnification (NMDA-NR1 - Alexa Fluor 488 green fluorescent, λ_excitation_ = 488 nm/λ_emission_ = 515 nm; PI red fluorescent, 535/617 nm).

### Gastric acid output study protocols

The method of isolated stomach perfusion by Ghosh and Shild^50^ was employed. Gastric acid output was investigated in acute experiments on 455 Wistar female rats. Food was withheld 24 hr before the experiment, but animals had free access to water. The rats were anesthetized with urethane (Sigma Aldrich, USA) at a dose of 1.1 g/kg intraperitoneally (i.p.). After the experiments, the animals were sacrificed via a urethane lethal dose of 3 g/kg. All of the data are representative of eleven independent experiments (*N* = 11) for each experimental group.

First, the anesthetized animals were tracheotomized to allow free passage of air to the lungs during stomach perfusion. Then, an incision was cut along the white line of the abdominal cavity to pull out the stomach pro tempore for removing food remains and further procedures. Catheters were inserted into the stomach lumen through the esophagus and duodenum. A peristaltic pump perfused saline solution (pH 7.0, 37 °C) through the esophagus catheter at a constant speed of 17 ml/10 min. Below the pyloric sphincter, a ligature was used to mount the catheter inserted through the incision into the duodenum. The output catheter was joined to the fraction collector. Next, the stomach was allowed to equilibrate for 1 hr. For the experiments, the fraction collector automatically collected serial perfusate samples every 10 min for a total collection period of approximately 2 hr.

The HCl acid concentration in the perfusate was quantified by the back-titration method using an ion meter. A quantity of 0.01 N NaOH, used for titration of one 10-min-sample up to pH 7.0, was equal to the HCl output in back-titration units. The debit of hydrochloric acid was expressed in titration units, listed in micromole (μmol/120 min). To calculate the HCl output that lasted 2 hr, all debits of 10-min-samples were considered.

For every rat, after the first 2 hr of the basal acid output measurements, GAS stimulants/secretagogues were injected and the stimulated acid output was also measured for 2 hr. The effect of secretagogues was evaluated by a statistically significant difference in relation to the basal acid secretion output. Following the same protocol, NMDA action on basal GAS was evaluated. For the investigation of NMDA action on the background of secretagogue-induced GAS, NMDA was injected 30–60 min after secretagogues injection, and output measurements lasted 2 hr. The study of NMDA action was also conducted under receptor blockade by selective antagonists, with the following injection sequence: (i) carbachol after control basal GAS measurements, (ii) inhibitor 40 min later, and (iii) NMDA 10 min after the inhibitor injection. The effects of antagonists and NMDA were evaluated by a statistically significant difference in relation to the secretagogue output.

### Bilateral cervical vagotomy

In rats under deep anesthesia, the trachea was released from muscle tissues in the lower part of the neck, and a midline abdominal incision was made for free air access. A tracheal tube was inserted through the incision into the trachea. After that, the esophagus was exposed by carefully retracting the liver toward the right. Then, ventral branches of the vagus nerve on the esophagus were confirmed and dissected. The HCl output measurements started 1 hr after vagotomy.

### Drugs and their injection protocols

In the study of NMDA influence on stimulated GAS the following secretagogues were used, being injected i.p., intravenous (i.v.) and intraduodenal (i.d.): carbachol (10 µg/kg, i.p.), histamine (3 mg/kg, i.v.), pentagastrin (0.26 mg/kg, i.p.), cytisine (0.34 mg/kg, i.d., Sopharma, Bulgaria), neutral monocomponent human insulin Acrtapid MC (1.2 u/kg, i.v.), 2-deoxy-D-glucose (100 mg/kg, i.v.) from Sigma Aldrich, USA. The GAS blockers, pentamine (3.2 µg/kg, i.p.), atropine (1 g/kg, i.p.), gastrozepin (3 mg/kg, i.p.), proglumide (10 mg/kg, i.p.), and ranitidine (2 mg/kg, i.p.) were used at doses offered by the manufacturers; AP5 (50 µg/kg, i.p.) (Sigma Aldrich, USA). We used NMDA as an agonist of NMDARs (3 mg/kg, i.p.)^27^ (Sigma Aldrich, USA). Prior to injections, the drugs were dissolved in 0.9% saline and then heated to 37 °C.

### Statistical analysis

All of the data were subjected to statistical analysis. The Shapiro-Wilks test was used for analysis of the data distribution type. As our data were normally distributed, Student’s t-test was used. The difference between two means was considered to be statistically significant when *P* was less than 0.05 (Figure symbols are *P < 0.05, **P < 0.01, ***P < 0.001). The results are expressed as the mean ± SD.
